# The strength of genetic interactions scales weakly with mutational effects

**DOI:** 10.1186/gb-2013-14-7-r76

**Published:** 2013-07-26

**Authors:** Andrea Velenich, Jeff Gore

**Affiliations:** 1 Department of Physics, Massachusetts Institute of Technology, 77 Massachusetts Avenue, Cambridge, MA 02139, USA

**Keywords:** Epistasis, Evolution, Fitness landscapes, Genetic interactions, Yeast

## Abstract

**Background:**

Genetic interactions pervade every aspect of biology, from evolutionary theory, where they determine the accessibility of evolutionary paths, to medicine, where they can contribute to complex genetic diseases. Until very recently, studies on epistatic interactions have been based on a handful of mutations, providing at best anecdotal evidence about the frequency and the typical strength of genetic interactions. In this study, we analyze a publicly available dataset that contains the growth rates of over five million double knockout mutants of the yeast *Saccharomyces cerevisiae*.

**Results:**

We discuss a geometric definition of epistasis that reveals a simple and surprisingly weak scaling law for the characteristic strength of genetic interactions as a function of the effects of the mutations being combined. We then utilized this scaling to quantify the roughness of naturally occurring fitness landscapes. Finally, we show how the observed roughness differs from what is predicted by Fisher's geometric model of epistasis, and discuss the consequences for evolutionary dynamics.

**Conclusions:**

Although epistatic interactions between specific genes remain largely unpredictable, the statistical properties of an ensemble of interactions can display conspicuous regularities and be described by simple mathematical laws. By exploiting the amount of data produced by modern high-throughput techniques, it is now possible to thoroughly test the predictions of theoretical models of genetic interactions and to build informed computational models of evolution on realistic fitness landscapes.

## Background

Genetic interactions [1] have shaped the evolutionary history of life on earth. They have been found to limit the accessibility of evolutionary paths [2], to confine populations to suboptimal evolutionary states and, on larger time scales, to control the rate of speciation [3]. Epistatic interactions can also be relevant to the development of complex human diseases such as diabetes [4]. Complex traits and diseases are determined by a multiplicity of genomic loci [5], whose independent effects and interactions [6] are often necessary to understand the phenotype of interest. Despite the broad implications of epistatic interactions, a quantitative characterization of their typical strength is still lacking. In this study, we consider growth rate in yeast as an example of a complex trait modulated by genetic interactions.

Previous studies [7-10] on the relation between the growth effects of a mutation and its epistatic interactions have often been based on a handful of mutations, and only in recent years has anecdotal evidence started being replaced by robust statements based on large data sets. Perhaps the most impressive of these datasets is the one made publicly available with the publication of the article entitled 'The genetic landscape of a cell' by Costanzo *et al. *[11]. The genome of the budding yeast *Saccharomyce cerevisiae *includes approximately 6,000 genes, about 1,000 of which are essential. Viable mutants can be constructed by knocking out any of the approximately 5,000 non-essential genes, by reducing the expression of the essential genes, or by partially compromising the functionality of the gene products. The dataset (see Additional file 1, Figure S1) has been compiled with the growth rates of about 5.4 million double knockout mutants, a sizable fraction of all possible double knockout mutants in yeast. Supported by the Costanzo *et al*. dataset, we consider the fundamental question of whether mutations with larger effects have stronger genetic interactions.

## Results and discussion

### An unbiased definition of genetic interactions

A basic approach to study genetic interactions is to consider two mutations with known effects on a quantitative trait, and to measure their combined effect in the double mutant [12]. Given [11,13] the growth rates of a wild type *S. cerevisiae *strain (g_00 _= 1) and of two single knockout mutants (g_01 _and g_10_), the growth rate of the double knockout mutant (g_11_) is adequately predicted by a multiplicative null model:

$${\text{g}}_{\text{11}}/{\text{g}}_{00}=\left({\text{g}}_{0\text{1}}/{\text{g}}_{00}\right)\left({\text{g}}_{\text{1}0}/{\text{g}}_{00}\right).$$

**Figure 1:**
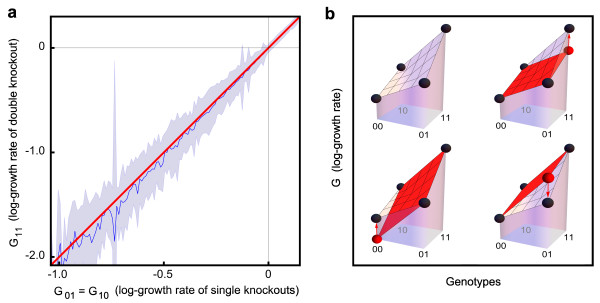
**The log growth rates of two mutations combine additively**. **(a) **The average effect of a double knockout (G_11_) as a function of the effects of the single knockouts (G_01 _and G_10_) is G_11 _= G_01 _+ G_10_. Experimental mean +/- standard deviation (blue line and blue shaded area) and prediction of the additive null model (red line). **(b) **Given two mutations, there are four possible mutants with their corresponding log growth rates (black dots). If three of the four log growth rates are known, the fourth one can be predicted by a linear extrapolation (red plane), and epistasis can be defined as the linear deviation from such prediction (red arrow). The magnitude of the deviation is the same regardless of which three of four mutants are chosen.

Equivalently, defining 'log growth' as the logarithm of the relative growth rate,

$$\text{G}=\text{lo}{\text{g}}_{\text{2}}\left(\text{g}/{\text{g}}_{00}\right),$$

the log growth of the double knockout mutant is predicted by an additive null model (Figure 1a):

$${\text{G}}_{\text{11}}={\text{G}}_{0\text{1}}+{\text{G}}_{\text{1}0}.$$

Epistatic interactions are identified as deviations from the null model, but several non-equivalent alternatives exist for quantifying these deviations [14]. The most common definition of epistasis considers the difference between the measured and the predicted growth rates for the double knockout mutant [11]:

$$e=\frac{g_{\text{11}}}{g_{\text{00}}}-\frac{g_{\text{01}}}{g_{\text{00}}}\frac{g_{\text{10}}}{g_{\text{00}}}$$

Importantly, this definition of *e *subtly constrains the possible values of epistasis. In fact, when combining very deleterious mutations, *e *cannot be large and negative even when the double knockout mutant is a synthetic lethal mutant:

$$\begin{array}{c} e=0-\left({\text{g}}_{0\text{1}}/{\text{g}}_{00}\right)\left({\text{g}}_{\text{1}0}/{\text{g}}_{00}\right)\approx 0,\\ \text{if}\;{\text{g}}_{0\text{1}}<<{\text{g}}_{00}\;\text{and}\;{\text{g}}_{\text{1}0}<<{\text{g}}_{00}. \end{array}$$

In order to avoid *a priori *constraints on the intensity of epistasis, genetic interactions can be defined as the ratio between the measured and predicted relative growth rates, leading to:

$$E=\text{lo}{\text{g}}_{\text{2}}\frac{g_{\text{11}}}{g_{\text{00}}}-\text{lo}{\text{g}}_{\text{2}}\frac{g_{\text{01}}}{g_{\text{00}}}-\text{lo}{\text{g}}_{2}\frac{g_{\text{10}}}{g_{\text{00}}}.$$

As an example, *E *= +1 indicates a double mutant whose growth rate is twice as large as would be expected based upon the multiplicative null model, whereas *E *= -1 indicates a double mutant whose growth rate is half as large as predicted. This definition of epistasis as fold deviation in the multiplicative model for growth rates is equivalent to a natural definition of epistasis as linear deviation in the additive model for log growth rates (Figure 1b):

$$E=\left(G_{00}+G_{11}\right)-\left(G_{01}+G_{10}\right)=\left(G_{11}-G_{01}-G_{10}\right).$$

A second bias of the common definition of epistasis is that *e *depends on the choice of which genotype is labeled as 'wild type' or '00', a choice which is always arbitrary, but more obviously so when studying engineered organisms or populations evolving in alternating environments [15]. By contrast,

$$\left|E\right|=\left|\left({\text{G}}_{00}+{\text{G}}_{\text{11}}\right)-\left({\text{G}}_{0\text{1}}+{\text{G}}_{\text{1}0}\right)\right|$$

depends only on which pair of genes is considered, being a geometric measure for the 'curvature' of the fitness landscape (Figure 1b).

The definition of *E *has found some favor in the theoretical literature [7,16], but it is not routinely used to analyze experimental data apart from rare exceptions [8,17]. Its main drawback is that synthetic lethals have a log growth rate of -∞, and require a separate although simpler analysis in which lethal interactions can simply be counted. The definition of *E *proves instead to be extremely valuable when quantifying the strength of non-lethal genetic interactions.

### Epistatic interactions scale weakly with mutational effects

With the appropriate definition of epistasis, a simple relation between the growth rate effects of two mutations and the expected strength of their interaction emerges.

**Figure 2:**
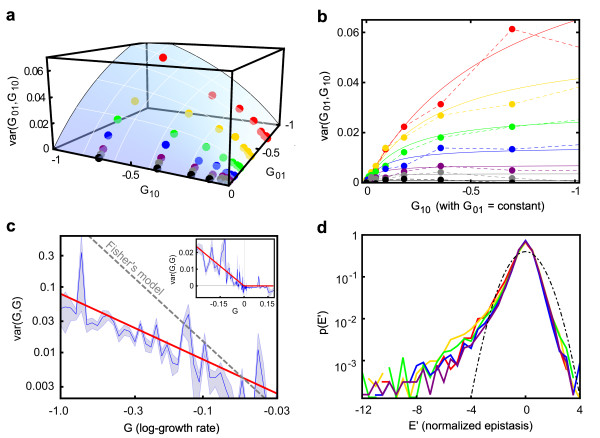
**The strength of epistatic interactions scales with the log growth effects of the interacting knockouts**. **(a) **Each dot represents the variance of several thousand epistatic interactions binned according to the log growth effects of the two single knockouts, G_01 _and G_10_. The blue surface is the phenomenological fit:
$\text{var}\left({\text{G}}_{0\text{1}},{\text{G}}_{\text{1}0}\right)=0.0\text{79}\times \text{2}\left|{\text{G}}_{0\text{1}}\right|\left|{\text{G}}_{\text{1}0}\right|/\left(\left|{\text{G}}_{0\text{1}}\right|+|{\text{G}}_{\text{1}0}|\right).$**(b) **Slices of the plot in (a) for G_01 _= constant. The dots are the same as in (a), and the solid lines represent the corresponding slice of the one-parameter fitting surface. **(c) **Diagonal slice of the plot in (a) with finer bins (G_01 _= G_10 _within 20%, G = mean(G_01_, G_10_)). The blue shaded area is the 25 to 75% confidence interval computed by bootstrap; the red line (var(G, G) = 0.079 G) is computed from the phenomenological model, and the dashed gray line, for which var(G, G) is proportional to G^2^, represents the lower bound to the slope predicted by the Fisher's geometric model. **(c, inset) **The epistatic interactions between beneficial mutations are vanishingly small, independently of the effect of the combined mutations. **(d) **Probability density functions p(E') for the strength of genetic interactions between two deleterious knockouts with similar log growth effects. Different colors correspond to knockouts with different effects: the growth rates effects of the single knockouts being combined are close to -38% (red), -22% (yellow), -12% (green), -6% (blue), and -3% (purple). Each curve has been rescaled so that all distributions have a standard deviation = 1. The left tail of the distributions displays a fat tail, describing the occurrence of strong negative genetic interactions (for comparison, the dashed-dotted black line is a normal distribution).

Let us consider two groups of mutations; in the first group, all mutations have log growth effect G_01_, and in the second group, all mutations have log growth effect G_10_. We can then build all possible double mutants obtained by combining one mutation from each group. In the absence of epistasis, all the double mutants have a log growth rate

$${\text{G}}_{\text{11}}={\text{G}}_{0\text{1}}+{\text{G}}_{\text{1}0},$$

and the distribution of genetic interactions is sharply peaked at *E *= 0. When epistasis is present, the distribution of genetic interactions has, in general, non-zero mean and standard deviation. Experimentally, however, the mean of genetic interactions is close to zero (this is why the null model remains approximately valid) (Figure 1a; Figure 2d). Even when the mean interaction is vanishing, the difference between the experimental dataset and the ideal case without interactions can be quantified by the finite value of the experimental standard deviation σ(G_01_, G_10_), which provides a numerical estimate for the characteristic strength of epistatic interactions.

In order to produce reliable numerical results, thousands of growth rates are necessary to characterize the probability distribution of epistasis. We analyzed the Costanzo *et al*. dataset by binning pairs of mutations according to the log growth effects of their single knockouts G_01 _and G_10_, using the method described above to outline the probability distribution of epistasis. We chose bin sizes that grow exponentially with G in order to ensure an approximately constant number of data points in each bin (see Materials and Methods; see Additional file 1, Figure S2). Most bins contain from thousands to tens of thousands of data points. For each bin, we computed

$$\text{var}\left(E\left({\text{G}}_{0\text{1}},{\text{G}}_{\text{1}0}\right)\right),$$

that is, the variance of the random variable *E *relative to the bin labeled by growth rates G_01 _and G_10_. In the rest of the paper we will refer to such variance as var(G_01_, G_10_), emphasizing that the variance in the strength of epistatic interactions is, eventually, a function of G_01 _and G_10 _(Figure 2a). The square root of the variance, σ(G_01_, G_10_), then represents the expected strength of epistasis as a function of the independently varying effects of the two single knockouts. A natural expectation for the dependence of epistasis on the effect of the combined mutations comes from rescaling Figure 1a; if all the log growth effects of single and double knockouts increase by a factor of two, then the strength of epistasis should also increase by a factor of two. Unexpectedly, however, when combining deleterious mutations, the strength of epistatic interactions does grow with the effects of the mutations that are combined, but the dependence is much weaker; when the effect of both single knockouts is doubled, the strength of epistasis increases only by a factor of √2 (Figure 2).

In more detail, we observed that if the effect of the first knockout (G_01_) is held constant, the dependence of the variance of epistasis on the effect of the second knockout (G_10_) is well approximated by a Michaelis-Menten law (Figure 2b):

$$\text{var}\left(G_{10}\right)=v\frac{\left|G_{\text{10}}\right|}{K+|G_{\text{10}}|}.$$

When the effects of both knockouts are free to vary, the requirement that the variance is a symmetric function of its two variables, *G*_01 _and *G*_10_, implies that K = |G_01_| and that *v *is proportional to G_01_. A one-parameter function which fits the seen variance over the whole range of deleterious fitness effects (Figure 2a) is then:

$$\text{var}\left(G_{\text{01,}}G_{\text{10}}\right)=\text{2c}\frac{\left|G_{\text{01}}G_{\text{10}}\right|}{\left|G_{\text{01}}|+|G_{\text{10}}\right|}\text{with }c=\text{0.079}.$$

This functional form can also be obtained from a simple model based on diffusion in fitness space (see Additional file 1, Supplementary text 1). An even simpler phenomenological fit, although slightly less accurate, is:

$$\text{var}\left({\text{G}}_{01},{\text{G}}_{10}\right)=\text{c}\surd \left(\left|{\text{G}}_{0\text{1}}\right|\left|{\text{G}}_{\text{1}0}\right|\right)$$

(see Additional file 1, Figure S3). Importantly, these functions capture two major features of the data; first, epistasis vanishes when G_01 _or G_10 _= 0; second, when the effects of the two knockouts are similar (G_01 _= G_10 _= G along the diagonal of the surface in Figure 2a), the variance of epistasis is approximately proportional to G (Figure 2c):

$$\text{var}({\text{G}}_{0\text{1}},{\text{G}}_{\text{1}0})=\text{c}\left|\text{G}\right|.$$

The scaling described above is seen only for deleterious knockouts. When combining the beneficial knockouts in the dataset instead, the strength of epistasis is close to zero (Figure 2c, inset). This might be because the slightly beneficial knockouts are not adaptive mutations, but simply remove genes that are not needed in the conditions chosen for the experiment, so that their interactions are likely to be negligible. However, in apparent contrast to this observation, recent studies [8,18] on adaptive mutations in *Escherichia coli *suggest that genetic interactions between adaptive mutations are mostly negative. In fact, during adaptation, the prevalence of negative interactions is likely to be caused by biased sampling, because the mutations that fix in the population are likely to be the ones that solve environmental or biological challenges for an organism. Diminishing returns arise because the appearance of multiple 'solutions' to the same challenge is not necessarily preferable over the presence of a single solution. Rather than focusing on mutations that fix during a bout of adaptation, the Costanzo *et al*. dataset includes a large fraction of all possible pairs of genes in the yeast genome. Because for most pairs the two genes are involved in unrelated biological processes, interactions are often vanishingly small. We did observe, however, that the distribution of epistatic interactions is asymmetric, with a heavy tail of deleterious interactions (Figure 2d).

### Experimental uncertainty generates spurious epistatic interactions

When inferring genetic interactions from experimental data, it is important to take into account that each measured growth rate is affected by some uncertainty, and that measurement errors in the growth rates could erroneously be interpreted as genetic interactions. Importantly, for each single and double mutant, the Costanzo *et al*. dataset provides the mean growth rate together with its estimated experimental uncertainty (the growth rate of each mutant being measured at least four times).

**Figure 3:**
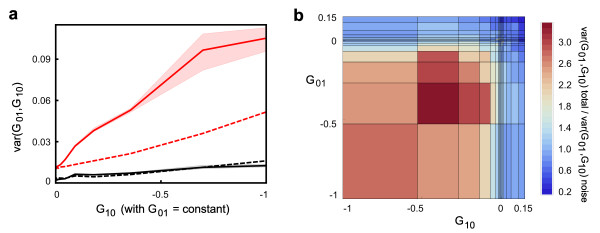
**Experimental noise does not account for all of the observed variance of epistasis**. **(a) **Comparison of experimentally measured variance (solid lines; shaded areas: 25 to 75% confidence intervals) and variance caused by experimental noise (dashed lines). If one of the two mutations is neutral, noise accounts for all of the observed variance (black). When deleterious mutations are combined, noise accounts for less than half of the observed variance (red, G_01 _≈ -0.7). **(b) **Ratio between total observed variance and noise-generated variance as a function of the log growth of the knockouts being combined. For deleterious knockouts, the ratio can be significantly greater than 1.

In order to quantify the effect of the experimental uncertainty on the inferred epistatic interactions, we constructed a number of mock datasets, assuming that the null model without epistatic interactions described biology exactly. In these datasets, each single knockout had the same growth rate as in the original dataset, and each double knockout had a growth rate equal to the product of the relative growth rates of the corresponding single knockouts. We then randomized the mock datasets by shifting each growth rate by a random amount sampled from a Student's *t*-distribution, with width depending on the corresponding experimental uncertainty reported in the original dataset (see Additional file 1, Supplementary text 3). As expected, analysis of these 'noisy' datasets revealed some epistasis, clearly caused by our addition of experimental noise rather than by any biological mechanism. We found that for pairs involving beneficial or neutral mutations, the variance computed in the mock datasets was comparable to or even greater than the variance observed in the original dataset (Figure 3a, black curves; Figure 3b, blue regions). This fact provides an important internal control, suggesting that the experimental noise has not been underestimated. In spite of this, for pairs of knockouts with substantially deleterious effects, experimental noise accounted for less than half of the total observed variance, with the rest representing genuine biological interactions (Figure 3a, red curves; Figure 3b, red regions).

**Figure 4:**
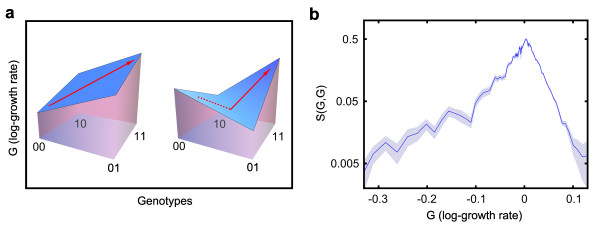
**Sign epistasis is less likely to occur between mutations with large effects**. **(a) **Examples of a smooth landscape with paths of monotonically increasing fitness (left) and a rugged landscape characterized by reciprocal sign epistasis (right). **(b) **Experimentally measured probability of observing sign epistasis as a function of the log growth of two single knockouts with similar effects (G_01 _= G_10 _within 20%, G = mean(G_01_, G_10_)). The blue shaded area is the standard error of the mean computed by bootstrap.

We then decomposed the variance observed in the original dataset into a contribution produced by experimental uncertainty and a contribution of biological origin; the strength of epistatic interactions was finally computed as the square root of the biological part of the variance. For deleterious knockouts, the relative difference between epistasis computed from the raw data and from the data after subtracting the experimental noise was less than 30%, emphasizing the significant but not overwhelming contribution of experimental noise to the observed variability. Figure 2(a-c) represents the 'biological' part of the observed epistasis; before subtracting the contribution of the experimental uncertainty, the plots are qualitatively similar, but quantitatively slightly different (see Additional file 1, Figure S4). Importantly, because variances are additive, the estimated contribution of the experimental uncertainty to epistasis is largely independent of the choice of the statistical distribution used to model experimental uncertainty. In two instances, however, the unknown details of the full distribution of experimental noise are important; when outlining the distribution of epistatic interactions (Figure 2d) and when describing the probability to observe sign epistasis (Figure 4b). In those two figures, we plotted the raw data, and did not attempt to deconvolve the contribution of experimental uncertainty.

### Comparison between theory and experiment

The scaling of epistasis observed in the Costanzo *et al*. dataset (Figure 2) is in sharp contrast to the predictions of Fisher's geometric model [19], a popular model of epistasis in which genetic interactions emerge from geometry. As we saw, when the effects of the two knockouts are similar (G_01 _= G_10 _= G), the variance of epistasis is approximately proportional to G. By contrast, in the Fisher's model, the variance var(G, G) would grow faster than G^2 ^(Figure 2c; see Additional file 1, Supplementary text 2), a much stronger dependence than the linear dependence observed experimentally.

A concrete numerical example can highlight the importance of the weaker-than-expected scaling of epistasis described in this study. Let us consider two gene knockouts, each of which reduces the relative growth rate by 5%, from 1.0 to 0.95. According to the multiplicative null model, the growth rate of the double knockout will be approximately 0.95^2^, or approximately 0.90. The questions now are: What kind of deviations could be expected around 0.90? Would a growth rate of 0.85 be surprising? What about a growth rate of 0.50?. Let us use the analytic fit discussed in the previous section

$${\text{g}}_{0\text{1}}={\text{g}}_{\text{1}0}=0.\text{95,}$$

Then

$${\text{G}}_{0\text{1}}={\text{G}}_{\text{1}0}=\text{lo}{\text{g}}_{\text{2}}\left(0.\text{95}\right)=-0.0\text{74,}$$

$${\text{G}}_{\text{11}}={\text{G}}_{0\text{1}}+{\text{G}}_{\text{1}0}=-0.\text{148,}$$

and

$$\sigma \left({\text{G}}_{0\text{1}},{\text{G}}_{\text{1}0}\right)=0.0\text{76.}$$

A +/- one standard deviation interval for the growth rate of the double knockout is then

$$\left[{\text{2}}^{-0.\text{148 }-0.0\text{76}},{\text{2}}^{-0.\text{148 }+\;0.0\text{76}}\right]=\left[0.\text{86},0.\text{95}\right].$$

Notice that it is not unlikely that epistasis will cancel the effect of the second mutation, so that the growth rate of the double knockout mutant is greater than 0.95, that is, greater than the growth rate of either of the single knockout mutants.

Let us now consider two gene knockouts with stronger effects, each of which reduces the growth rate from 1.0 to 0.60. Then

$${\text{G}}_{0\text{1}}={\text{G}}_{\text{1}0}=\text{lo}{\text{g}}_{\text{2}}\left(0.\text{6}0\right)=-0.\text{737,}$$

about 10 times as large as the log growth of the single mutants in the previous example. The Fisher's model would predict a σ(G, G) at least 10 times larger than in the previous example (σ(G, G)≥0.76), and an interval of likely growth rates for the double knockout mutants at least as large as

$$\left[{\text{2}}^{-\text{ 1}.\text{47}-0.\text{76}},{\text{2}}^{-\text{1}.\text{47}+0.\text{76}}\right]=\left[0.\text{213},0.\text{61}0\right].$$

Notice how, once again, it is not unlikely that owing to genetic interactions, the growth rate of the double knockout mutant is greater than 0.60, the growth rate of either of the two single knockout mutants. The analytic model derived from the experimental data leads to a strikingly different conclusion:

$$\sigma \left({\text{G}}_{0\text{1}},{\text{G}}_{\text{1}0}\right)=0.\text{241,}$$

and the +/- one standard deviation interval for the growth rate of the double knockout becomes

$$\left[{\text{2}}^{-\text{1}.\text{47}-0.\text{241}},{\text{2}}^{-\text{1}.\text{47}+0.\text{241}}\right]=\left[0.\text{3}0\text{5},0.\text{425}\right].$$

In this case, a deviation from the null model that is greater than three standard deviations would be needed for the double knockout mutant to have a growth rate greater than that of the single knockout (0.60), making the event extremely unlikely.

### Epistasis constrains the evolutionary dynamics

The previous section provided two examples of reciprocal sign epistasis, realized when two deleterious mutations produce a double mutant that is fitter than either of the two single mutants (Figure 4a). In those cases, a fitness valley limits the evolutionary accessibility of the fitter double mutant, and only on longer time scales may the simultaneous appearance of two mutations [20,21] drive a population to the new local fitness maximum. In this context, the scaling behavior of epistasis is of great importance, because it determines the number and the topology of the evolutionarily accessible paths [2,22,23], ultimately affecting the possible outcomes of the evolutionary process.

In order to describe how epistasis shapes the naturally occurring fitness landscapes, let us consider S(G, G), the probability to observe sign epistasis when combining two mutations with similar growth rate effects, G. Here, S(G, G) depends on the typical interaction strength,

$$\sigma \left(\text{G},\text{G}\right)=\surd \text{var}\left(\text{G},\text{G}\right).$$

In particular, if σ(G, G) is proportional to G, then the probability of observing sign epistasis is independent of G. The Fisher's model implies a super-linear dependence of σ(G, G) on G, thus predicting a greater probability of observing sign epistasis among mutations with strong effects. Instead, if the scaling of σ(G, G) is proportional to √G (Figure 2), then sign epistasis is more likely to occur among mutations with small effects (Figure 3b). When the relative growth rate effects of the single knockouts are small (<2 to 3%), experimental uncertainty prevents us from pinpointing which pairs of genes are epistatic. This does not mean, however, that mutations with small effects do not interact. Assuming that the scaling of epistasis we measured directly for mutations with intermediate and large effects extends to mutations with small effects, a consequence of the observed scaling of epistasis is the roughening of the local fitness landscape in the proximity of an evolutionary optimum; when the fitness effects of available mutations become small [24], epistatic interactions become increasingly relevant [25,26], reducing the accessibility of evolutionary paths and further slowing down the rate of adaptation [27,28]. The evolutionary dynamics on correlated fitness landscapes [10,29] with the realistic correlations described here certainly deserves further experimental and theoretical investigation.

### The scaling of genetic interactions may be generic

**Figure 5:**
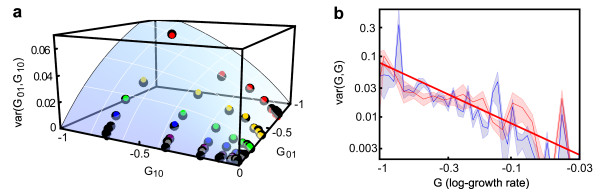
**Point mutations have similar epistatic interactions to those of entire gene knockouts**. **(a) **Comparison between the variance observed in double gene knockout mutants (rainbow dots, same as in Figure 2a) and the variance observed in mixed double mutants generated by combining a gene knockout with point mutations in a different gene (black dots). **(b) **The red curve is the diagonal slice of the plot in (a) (G_01 _= G_10 _within 20%, G = mean(G_01_, G_10_)), and the red shaded area is the 25 to 75% confidence interval for the mixed double mutant variance. For comparison, the blue curves describe the variance for double gene knockouts as in Figure 2c. As in Figure 2c, the red line has equation var(G, G) = 0.079 G.

To date, our analysis has been limited to interactions between entire gene knockouts. Although mutations with extreme effects on gene regulation and horizontal gene transfer are biologically relevant mechanisms for the removal or acquisition of whole genes at once, organisms explore possible genetic variants largely through the accumulation of single point mutations. The Costanzo *et al*. data et contains thousands of double mutants for which the first mutation is a gene knockout and the second mutation consists of one or more point mutations in a different gene, causing the gene product to misfold in a temperature-sensitive way. Although the distribution of growth rate effects for point mutations is different than for single gene knockouts (see Additional file 1, Figure S2), the statistics of genetic interactions are remarkably similar when combining two single knockouts and when combining a single knockout with a point mutation (Figure 5). A similar scaling is also seen for the epistatic interactions between single gene knockouts and decreased abundance by mRNA perturbation [30] (DamP) perturbations of a second gene (see Additional file 1 Figure S5). The analysis of these hybrid double mutants suggests that the statistics of the interactions between any two genetic perturbations are determined only by their growth rate effects [31], and not by their biological origin in terms of point mutations or gene knockouts.

### A comparison between different definitions of epistasis

**Figure 6:**
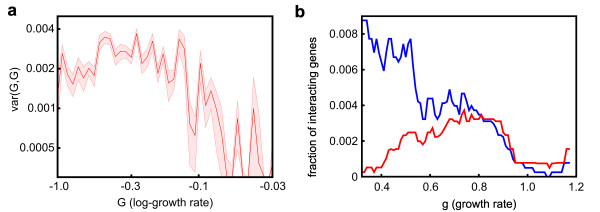
**Comparison between the traditional and the geometric definitions of epistasis (*e *and *E*, respectively)**. **(a) **Figure equivalent to Figure 2c, using the traditional definition of epistasis. **(b) **The fraction of genes interacting with a specific gene is a function of the growth rate effect of such gene. Only the 10,000 most interacting pairs the geometric definition (blue) and the traditional definition (red) are considered to be interactions.

Importantly, any quantitative result on epistasis is a consequence of how epistasis is defined. Of particular interest is how strong an epistatic interaction is deemed to be, based upon its ranking when compared with that of other pairs of mutations. Although the 'traditional' definition

$$e=g_{11}/g_{00}-\left(g_{01}/g_{00}\right)\left(g_{10}/g_{00}\right)$$

and the 'geometric' definition

$$E={\text{G}}_{\text{11}}-\left({\text{G}}_{0\text{1}}+{\text{G}}_{\text{1}0}\right)$$

agree about the sets of positive and negative interactions, they assign different strengths and, more importantly, different rankings to the same pair of interacting mutations. As an example, if the Costanzo *et al*. dataset is analyzed using the 'traditional' definition of genetic interactions, then the linear dependence of var(G, G) on G in Figure 2c is replaced by an oddly non-monotonic dependence, displaying weaker interactions for pairs of genes with either very small or very large fitness effects (Figure 6a). As mentioned previously, this decrease in the inferred strength of epistatic interactions for very deleterious mutations is a mathematical consequence of the traditional definition of epistasis, rather than a property of genetic interactions. The same bias would lead us to conclude that genes with strong effects on growth are almost non-interacting (Figure 6b, red line). However, because previous studies have determined that essential genes partake in more interactions than do non-essential genes [32], it is also reasonable to expect that non-lethal genes with large growth effects are involved in more interactions than genes with small growth effects. Indeed, according to the 'geometric' definition of epistasis, the fraction of genes with which a gene interacts steadily increases with the growth rate effect of the gene (Figure 6b, blue line). By contrast, the traditional definition of epistasis, consistently assigns low rankings to interactions between genes with large growth rate defects, as confirmed by a further analysis comparing the two definitions of epistasis against interactions inferred from the Gene Ontology (GO) database [33] (see Additional file 1, Figure S6). According to the geometric definition of epistasis, genetic networks [34] are denser than expected not only among essential gene [32], but also among genes with large growth effects.

Finally, it is important to emphasize that the traditional definition of epistasis remains slightly more successful at discovering the functional relations between genes, as cataloged in the GO database (see Additional file 1, Figure S6). Part of the reason for this could be that some of those functional characterizations were suggested by the traditional definition of epistasis in the first place. It is certainly true, however, that many of the top-ranking interactions according to the geometric definition of epistasis involve single and double mutants with small growth rates; for those mutants, experimental noise is relatively large, and this may cause a few weakly interacting pairs to be incorrectly ranked as strongly interacting. It is likely that the experimental protocols could be easily adjusted to reduce the relative uncertainty on the growth rate of especially slow-growing mutants to avoid this issue (for example, by allowing for a much longer time for growth or by measuring the growth rates of additional replicates).

### Conclusions

We analyzed the growth rates of about five million double mutants in the dataset associated with the work by Costanzo *et al*. We characterized how the strength of genetic interactions depends on the growth effects of the mutations being combined, and found a weaker dependence than that predicted by current theoretical models. Although the results were obtained mainly from entire gene knockouts, there is some evidence that the observed scaling might extend to the interactions between single point mutations. The scaling of epistasis might or might not be generic [35,36]; important drivers could be the harshness of the environment [37], details about the evolutionary history [38-40], sexual versus asexual reproduction [41] and, perhaps most importantly, metabolic [42-45] and genetic complexity [46,47]. In general, the experimentally observed scaling suggests a previously unexplored class of correlated fitness landscapes with tunable roughness, in which epistasis depends explicitly on the effects of the mutations being combined.

A clear limitation of our discussion is that only pair interactions were considered. Although high-throughput experiments will provide data on higher-order interactions, a solid understanding of pair interactions remains necessary before addressing n-mutation interactions. A genuine three-mutation interaction, for instance, should be defined as the unexplained deviation from what can be computed by combining the effects of all relevant mutations and their pair interactions [10,48], perhaps using linear fits within the additive null model for log growth rates.

The results we present here were based on a geometric definition of epistasis. We compared this definition with a more standard definition, highlighting the desirable mathematical properties of the geometric definition and the simple phenomenological relations it produces.

In conclusion, although each epistatic interaction between specific genes depends on biological details and remains largely unpredictable from first principles, we have shown that the statistical properties of an ensemble of interactions can display conspicuous regularities, and can be described by simple mathematical laws.

## Materials and methods

The Costanzo *et al*. dataset is publicly available [49]. The file http://sgadata_costanzo2009_rawdata_101120.txt.gz was downloaded on August 17, 2010 and analyzed with Mathematica (code available at the Gore laboratory website [50]). We restricted our analysis to double knockout mutants whose growth rates were positive numerical values and for which the growth rates of both single mutants were numerical values (see Additional file 1, Figure S1). Some genes appear in the dataset both as query and array genes; care was taken to avoid double counting.

The exponentially growing intervals used for the binning of the log growth rate effects were defined as [-2^n^, -2^n-1^] for an appropriate range of integer n's. Owing to the rarity of extremely deleterious mutations, bins for positive n's contained only a few data points, while bins with large negative n's were extremely small. In the figures we reported only bins for *n *= -7 to 0, containing log growth rate effects ranging from -2^0 ^= -1 to -2^-8 ^= -0.0039 or, alternatively, relative growth rate effects ranging from 2^-1 ^= 0.5 to 2^-0.0039 ^= 0.997. Different choices for the binning sizes and positions did not significantly alter the results of the analysis.

In order to quantify the contribution of experimental uncertainty to epistasis, we generated nine randomized mock datasets. The mean level of noise-generated epistasis in these nine datasets is reported in Figure 4 (dashed lines), and we provide an extensive discussion of the choice of Student's *t*-distributions to generate the mock datasets from the original dataset (see Additional file 1, Supplementary text 3).

The GO database go_201207-assocdb-tables.tar.gz was downloaded from the GO site [51] on July 19, 2012. The MySQL database was queried with Python and analyzed Mathematica (code available upon request).

## Abbreviations

DamP: Decreased abundance by mRNA perturbation; GO: Gene Ontology.

## Conflict of interest

The authors declare that they have no conflict of interest.

## Authors' contributions

AV and JG designed research; AV performed research and analyzed data; AV and JG wrote the paper. Both authors have read and approved the final manuscript.

## Supplementary Material

Additional file 1 **Supplementary Figures and Text**. Click here for file

## References

[B1] Phillips PC Epistasis - The essential role of gene interactions in the structure and evolution of genetic systems. Nat Rev Genet 2008 14 855 867 10.1038/nrg2452 18852697 PMC2689140

[B2] Weinreich DM Delaney NF DePristo MA Hartl DL Darwinian evolution can follow only very few mutational paths to fitter proteins. Science 2006 14 111 114 10.1126/science.1123539 16601193

[B3] Dettman JR Sirjusingh C Kohn LM Anderson JB Incipient speciation by divergent adaptation and antagonistic epistasis in yeast. Nature 2007 14 585 588 10.1038/nature05856 17538619

[B4] Hoh J Ott J Mathematical multi-locus approaches to localizing complex human trait genes. Nat Rev Genet 2003 14 701 709 12951571 10.1038/nrg1155

[B5] Mackay TFC Stone EA Ayroles JF The genetics of quantitative traits: challenges and prospects. Nat Rev Genet 2009 14 565 577 19584810 10.1038/nrg2612

[B6] Jansen RC Studying complex biological systems using multifactorial perturbation. Nat Rev Genet 2003 14 145 151 12560811 10.1038/nrg996

[B7] Gros PA Le Nagard H Tenaillon O The evolution of epistasis and its links with genetic robustness, complexity and drift in a phenotypic model of adaptation. Genetics 2009 14 277 293 10.1534/genetics.108.099127 19279327 PMC2674823

[B8] Khan AI Dinh DM Schneider D Lenski RE Cooper TF Negative epistasis between mutations in an evolving bacterial population. Science 2011 14 1193 1196 10.1126/science.1203801 21636772

[B9] Wilke CO Adami C Interaction between directional epistasis and average mutational effects. Proc R Soc Lond B Biol Sci 2001 14 1469 1474 10.1098/rspb.2001.1690 PMC1088765 11454290

[B10] Beerenwinkel N Pachter L Sturmfels B Elena SF Lenski R Analysis of epistatic interactions and fitness landscapes using a new geometric approach. BMC Evol Biol 2007 14 60 73 10.1186/1471-2148-7-60 17433106 PMC1865543

[B11] Costanzo M Baryshnikova A Bellay J Kim Y Spear ED Sevier CS Ding H Koh JLY Toufighi K Mostafavi S Prinz J St Onge RP VanderSluis B Makhnevych T Vizeacoumar FJ Alizadeh S Bahr S Brost RL Chen Y Cokol M Deshpande R Li Z Lin Z-Y Liang W Marback M Paw J San Luis B-J Shuteriqi E Tong AHY van Dyk N The genetic landscape of a cell. Science 2010 14 425 431 10.1126/science.1180823 20093466 PMC5600254

[B12] Dixon SJ Costanzo M Baryshnikova A Andrews B Boone C Systematic mapping of genetic interaction networks. Annu Rev Genet 2009 14 601 625 10.1146/annurev.genet.39.073003.114751 19712041

[B13] Baryshnikova A Costanzo M Kim Y Ding H Koh J Toufighi K Youn J-Y Ou J San Luis B-J Bandyopadhyay S Hibbs M Hess D Gingras A-C Bader GD Troyanskaya OG Brown GW Andrews B Boone C Myers CL Quantitative analysis of fitness and genetic interactions in yeast on a genome scale. Nat Methods 2010 14 1017 1024 10.1038/nmeth.1534 21076421 PMC3117325

[B14] Mani R StOnge RP Hartman JL Giaever G Roth FP Defining genetic interaction. Proc Natl Acad Sci USA 2008 14 3461 3466 10.1073/pnas.0712255105 18305163 PMC2265146

[B15] Tan L Gore J Slowly switching between environments facilitates reverse evolution in small populations. Evolution 2012 14 3144 3154 10.1111/j.1558-5646.2012.01680.x 23025604

[B16] Peters AD Lively CM Wolf JB, Brodie ED, Wade MJ Epistasis and the maintenance of sex. Epistasis and the evolutionary process 2000 Oxford: Oxford University Press 99 112

[B17] Martin G Elena SF Lenormand T Distributions of epistasis in microbes fit predictions from a fitness landscape model. Nat Genet 2007 14 555 560 10.1038/ng1998 17369829

[B18] Chou HH Chiu HC Delaney NF Segre D Marx CJ Diminishing return epistasis among beneficial mutations decelerates adaptation. Science 2011 14 1190 1192 10.1126/science.1203799 21636771 PMC3244271

[B19] Fisher RA The Genetical Theory of Natural Selection. 1930 Oxford: Clarendon Press

[B20] Weinreich DM Watson RA Chao L Sign epistasis and genetic constraint on evolutionary trajectories. Evolution 2005 14 1165 1174 16050094

[B21] Weissman DB Desai MM Fisher DS Feldman MW The rate at which asexual populations cross fitness valleys. Theor Pop Biol 2009 14 286 300 10.1016/j.tpb.2009.02.006 19285994 PMC2992471

[B22] Poelwijk FJ Kiviet DJ Weinreich DM Tans ST Empirical fitness landscapes reveal accessible evolutionry paths. Nature 2007 14 383 386 10.1038/nature05451 17251971

[B23] Velenich A Gore J Synthetic approaches to understanding biological constrains. Curr Opin Chem Biol 2012 14 323 328 10.1016/j.cbpa.2012.05.199 22682889 PMC3432407

[B24] Eyre-Walker A Keightley PD The distribution of fitness effects of new mutations. Nat Rev Genet 2007 14 610 618 17637733 10.1038/nrg2146

[B25] Tan L Serene S Chao HX Gore J Hidden randomness between fitness landscapes limits reverse evolution. Phys Rev Lett 2011 14 198102 21668204 10.1103/PhysRevLett.106.198102

[B26] Woods RJ Barrick JE Cooper TF Shrestha U Kauth MR Lenski RE Second-order selection for evolvability in a large Escherichia coli population. Science 2011 14 1433 1436 10.1126/science.1198914 21415350 PMC3176658

[B27] Orr HA The population genetics of adaptation the distribution of factors fixed during adaptive evolution. Evolution 1998 14 935 949 10.2307/2411226 28565213

[B28] Orr HA The genetic theory of adaptation a brief history. Nat Rev Genet 2005 14 119 127 15716908 10.1038/nrg1523

[B29] Kryazhimskiy S Tkčik G Plotkin JB The dynamics of adaptation on correlated fitness landscapes. Proc Natl Acad Sci USA 2009 14 18638 18643 10.1073/pnas.0905497106 19858497 PMC2767361

[B30] Breslow DK Cameron DM Collins SR Schuldiner M Stewart-Ornstein J Newman HW Braun S Madhani HD Krogan NJ Weissman JS A comprehensive strategy enabling high-resolution functional analysis of the yeast genome. Nat Methods 2008 14 711 718 10.1038/nmeth.1234 18622397 PMC2756093

[B31] Xu L Barker B Gu Z Dynamic epistasis for different alleles of the same gene. Proc Natl Acad Sci USA 2012 14 10420 10425 10.1073/pnas.1121507109 22689976 PMC3387062

[B32] Davierwala AP Haynes J Li Z Brost RL Robinson MD Yu L Mnaimneh S Ding H Zhu H Chen Y Cheng X Brown GW Boone C Andrews BJ Hughes TR The synthetic genetic interaction spectrum of essential genes. Nat Genet 2005 14 1147 1152 10.1038/ng1640 16155567

[B33] Barabási AL Oltvai ZN Network biology: understanding the cell's functional organization. Nat Rev Genet 2004 14 101 113 10.1038/nrg1272 14735121

[B34] The Gene Ontology Consortium Gene ontology: tool for the unification of biology. Nat Genet 2000 14 25 29 10.1038/75556 10802651 PMC3037419

[B35] Dixon SJ Fedyshyn Y Koh JLY Keshava Prasad TS Chahwan C Chua G Toufighi K Baryshnikova A Hayles J Hoe K-L Kim D-U Park H-O Myers CL Pandey A Durocher D Andrews BJ Boone C Significant conservation of synthetic lethal genetic interaction networks between distantly related eukaryotes. Proc Natl Acad Sci USA 2008 14 16653 16658 10.1073/pnas.0806261105 18931302 PMC2575475

[B36] Tischler J Lehner B Fraser AG Evolutionary plasticity of genetic interaction networks. Nat Genet 2008 14 390 391 10.1038/ng.114 18362882

[B37] Harrison R Papp B Pál C Oliver SG Delneri D Plasticity of genetic interactions in metabolic networks of yeast. Proc Natl Acad Sci USA 2007 14 2307 2312 10.1073/pnas.0607153104 17284612 PMC1892960

[B38] Wagner A Gene duplications robustness and evolutionary innovations. BioEssays 2008 14 367 373 10.1002/bies.20728 18348184

[B39] Wagner A Distributed robustness versus redundancy as causes of mutational robustness. BioEssays 2005 14 176 188 10.1002/bies.20170 15666345

[B40] Roguev A Bandyopadhyay S Zofall M Zhang K Fischer T Collins SR Qu H Shales M Park H-O Hayles J Hoe K-L Kim D-U Ideker T Grewal SI Weissman JS Krogan NJ Conservation and rewiring of functional modules revealed by an epistasis map in fission yeast. Science 2008 14 405 410 10.1126/science.1162609 18818364 PMC2753251

[B41] Azevedo RBR Lohaus R Srinivasan S Dang KK Burch CL Sexual reproduction selects for robustness and negative epistasis in artificial gene networks. Nature 2006 14 87 90 10.1038/nature04488 16511495

[B42] Segrè D DeLuna A Church GM Kishony R Modular epistasis in yeast metabolism. Nat Genet 2005 14 77 83 15592468 10.1038/ng1489

[B43] He X Qian W Wang Z Li Y Zhang J Prevalent positive epistasis in Escherichia coli and Saccharomyces cerevisiae metabolic networks. Nat Genet 2010 14 272 276 10.1038/ng.524 20101242 PMC2837480

[B44] Szappanos B Kovács K Szamecz B Honti F Costanzo M Baryshnikova A Gelius-Dietrich G Lercher MJ Jelasity M Myers CL Andrews BJ Boone C Oliver SG Pál C Papp B An integrated approach to characterize genetic interaction networks in yeast metabolism. Nat Genet 2011 14 656 662 10.1038/ng.846 21623372 PMC3125439

[B45] Almaas E Kovács B Vicsek T Oltvai ZN Barabási AL Global organization of metabolic fluxes in the bacterium Escherichia coli. Nature 2004 14 839 843 10.1038/nature02289 14985762

[B46] Sanjuán R Elena SF Epistasis correlates to genomic complexity. Proc Natl Acad Sci USA 2006 14 14402 14405 10.1073/pnas.0604543103 16983079 PMC1599975

[B47] Sanjuán R Nebot MR A network model for the correlation between epistasis and genomic complexity. PLoS One 2008 14 e2663 10.1371/journal.pone.0002663 18648534 PMC2481279

[B48] Wood K Nishida S Sontag ED Cluzel P Mechanism-independent method for predicting response to multidrug combinations in bacteria. Proc Natl Acad Sci USA 2012 14 12254 12259 10.1073/pnas.1201281109 22773816 PMC3409729

[B49] The Genetic Landscape of the Cell. http://drygin.ccbr.utoronto.ca/~costanzo2009

[B50] Gore Laboratory. http://www.gorelab.org/software.html

[B51] Gene Ontology. GO Database Downloads. http://www.geneontology.org/GO.downloads.database.shtml

