# Supplementary material for: The strength of genetic interactions scales weakly with mutational effects
Source: Genome Biol. 2013 Jul 26;14(7):R76. doi: 10.1186/gb-2013-14-7-r76 (PMC4053755; doi:10.1186/gb-2013-14-7-r76)
Supplement: Additional file 1 — Supplementary Figures and Text. [file gb-2013-14-7-r76-S1.PDF]

SUPPLEMENTARY INFORMATION
for
THE STRENGTH OF GENETIC INTERACTIONS SCALES WEAKLY WITH THE MUTATIONAL EFFECTS

Andrea Velenich [1] and Jeff Gore [1]
[1] Department of Physics, Massachsetts Institute of Technology, Cambridge, MA 02139

Figure S1

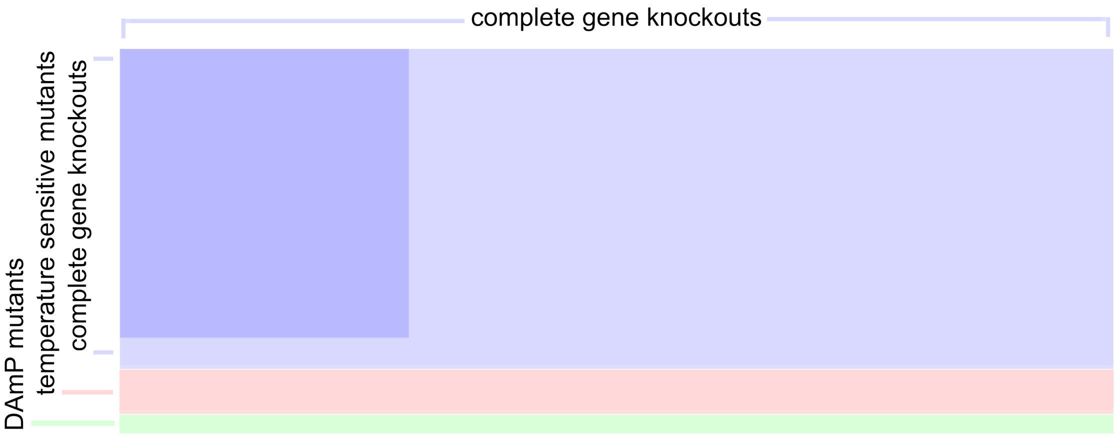

Figure S1 | The core of the DRYGIN data set is a 1712x3885 matrix whose entries are the growth rates of all possible double knockouts, obtained by crossing 1712 single knockouts of "query" genes with 3885 single knockouts of "array" genes. Since a few of the growth rates of the single knockouts mutants are reported as "NaN" (Not a Number), we restricted our analysis to a smaller 1570x3880 matrix, for which the growth rates of all single knockouts mutants are numerical values. Of the 1570 entries relative to query genes, 1287 are complete gene knockouts (blue rows); 191 are mutations which cause the gene product to misfold in a temperature-sensitive way (pink rows); 92 are DAmP mutants (Decreased Abundance by mRNA Perturbation) (green rows).

The darker blue square in the matrix represents double knockout mutants obtained from 1138 genes which appear both in the array and in the query subsets. For each pair of genes in this subset the double knockout mutants have been built twice, once as an "array x query" combination and once as a "query x array" combination. When both growth rates were numbers we used their average as the growth rate of the double knockout mutant. For about 5% of the pairs, one of the two combinations yielded "NaN", whereas the other one yielded a numerical growth rate; in those cases the numerical value was used as the growth rate of the double knockout mutant. For another 5% of the pairs, both growth rates were "NaN" and those pairs were dropped from the analysis. More generally, all double mutants with "NaN" and

negative growth rates have not been considered during the analysis, whereas every mutant with a positive growth rate, even if very close to zero, has been considered.

Figure S2

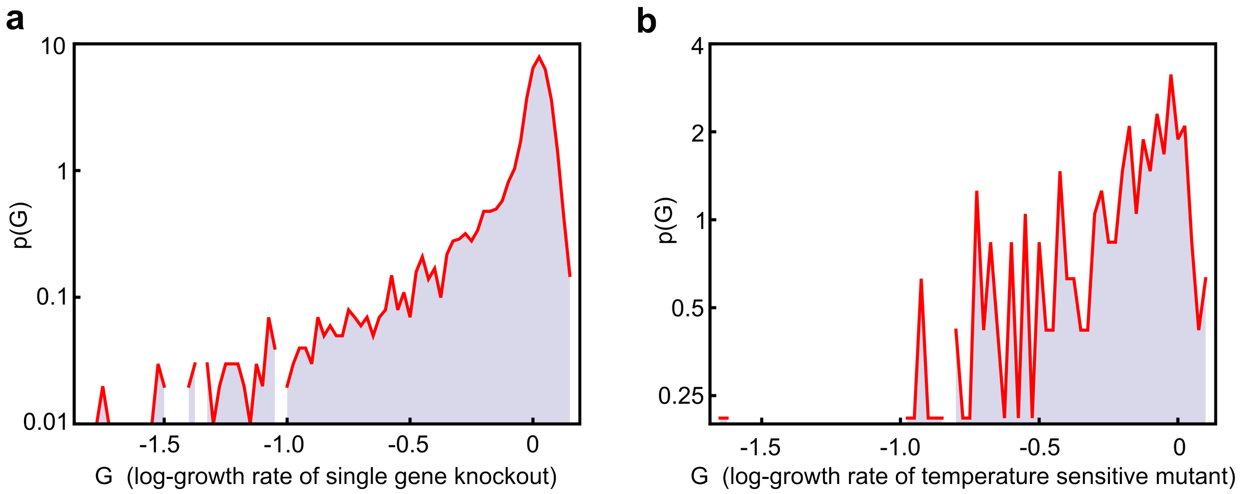

Figure S2 | Probability distributions p(G) of the log-growth effects for (a) all the single gene knockouts and (b) all the temperature sensitive mutants in the DRYGIN data set. The probability to observe a log-growth effect of G decreases approximately exponentially with |G|. Due to the scarcity of mutants with log-growth smaller than -1.0 and the consequent uncertainty in determining their statistical properties, the analysis in the main text considered only mutants for which G > -1.0 (corresponding to a growth rate of 0.5, relatively to the wild type).

Figure S3

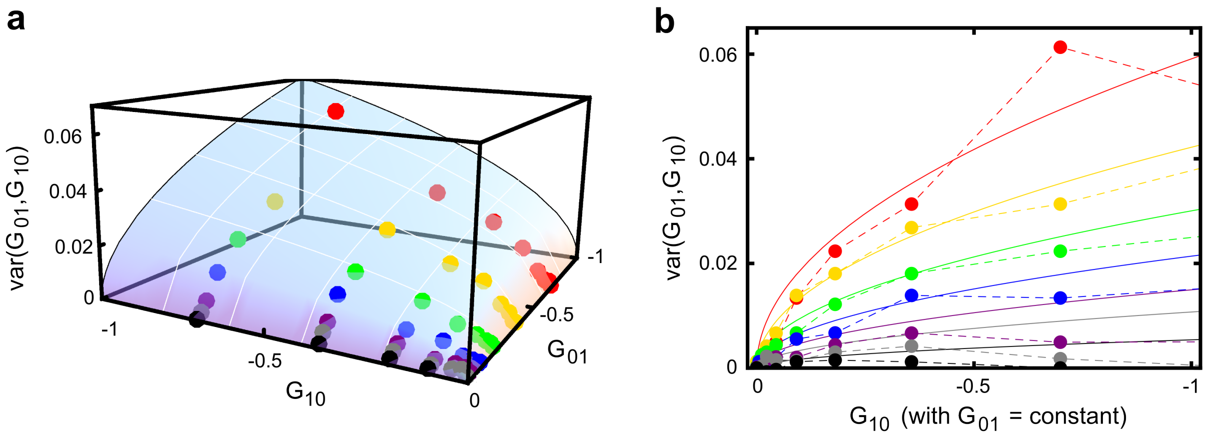

Figure S3 | The strength of epistatic interactions scales with the log-growth effects of the interacting knockouts. As in Figures 2a and 2b in the main text, each dot represents the variance of several thousand epistatic interactions binned according to the log-growth effects of the two single knockouts, G_{01} and G_{10}. Here we show how the data can be alternatively fit by a simple power law. (a) The blue surface is the phenomenological fit: var(G_{01}, G_{10}) = 0.071 Sqrt(|G_{01}|| G_{10}|). (b) Slices of the plot in (a) for G_{01} = constant. The dots are the same as in (a) and the solid lines represent the corresponding slice of the one-parameter fitting surface.

Figure S4

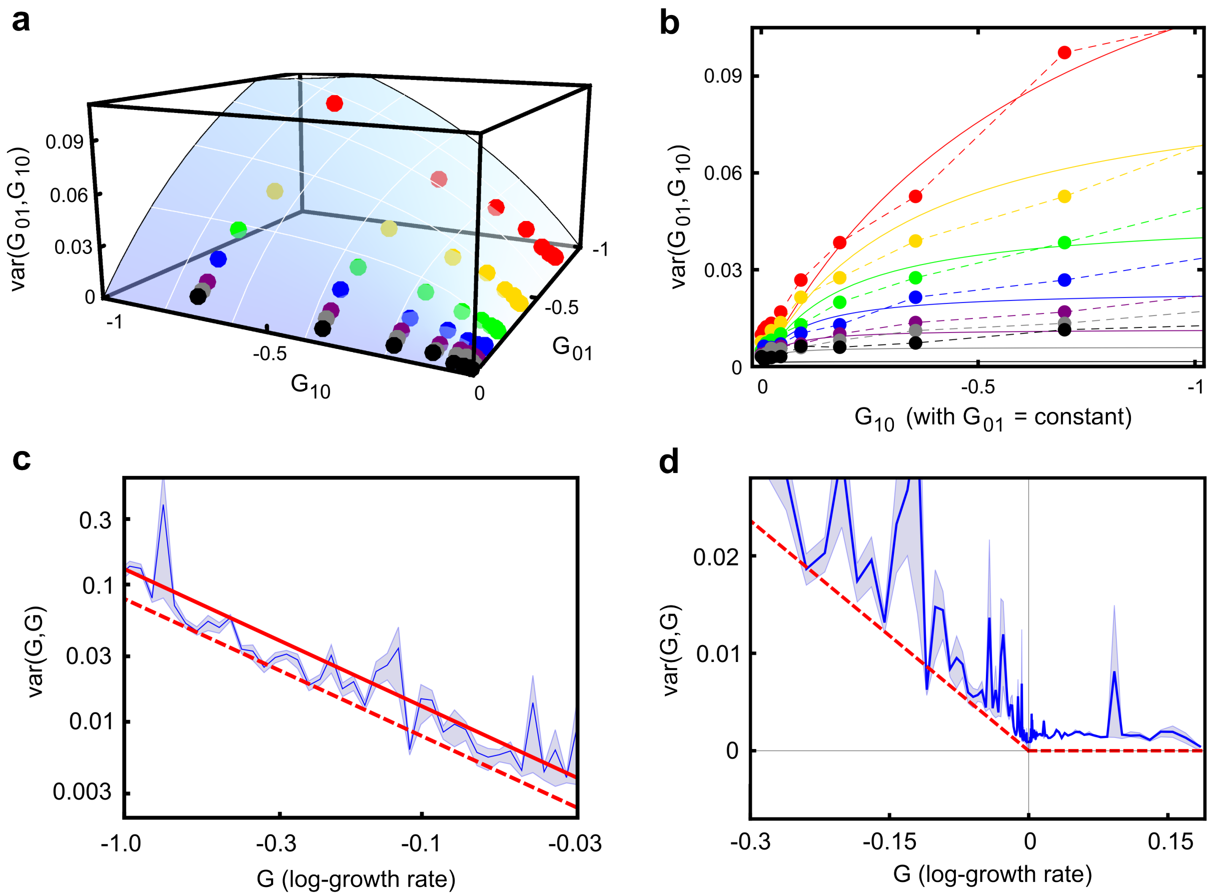

Figure S4 | The strength of epistatic interactions scales with the log-growth effects of the interacting knockouts. In contrast to Figures 2a, 2b, 2c and 2c Inset in the main text, here we plot the raw data, before subtracting the estimated contribution of the experimental noise. (a) Each dot represents the variance of several thousand epistatic interactions binned according to the log-growth effects of the two single knockouts, G_{01} and G_{10}. The blue surface is the phenomenological fit: var(G_{01}, G_{10}) = c x 2 |G_{01}||G_{10}| / (|G_{01}| + |G_{10}|). In this case c = 0.130, whereas after subtraction of experimental noise c = 0.079. (b) Slices of the plot in (a) for G_{01} =

constant. The dots are the same as in (a) and the solid lines represent the corresponding slice of the one-parameter fitting surface. (c) Diagonal slice of the plot in (a) with finer bins ($G_{01} = G_{10}$ within 20%, G = mean(G_{01}, G_{10})). The blue shaded area is the 25%-75% confidence interval computed by bootstrap; the solid red line (var(G, G) = 0.130 G) is computed from the phenomenological model and the dashed red line (var(G, G) = 0.079 G) is, for comparison, the corresponding line after subtracting experimental noise. (d) Diagonal slice of the plot in (a) showing both deleterious and beneficial knockouts. The dashed red lines are, for comparison, the fitting lines after subtracting experimental noise.

Figure S5

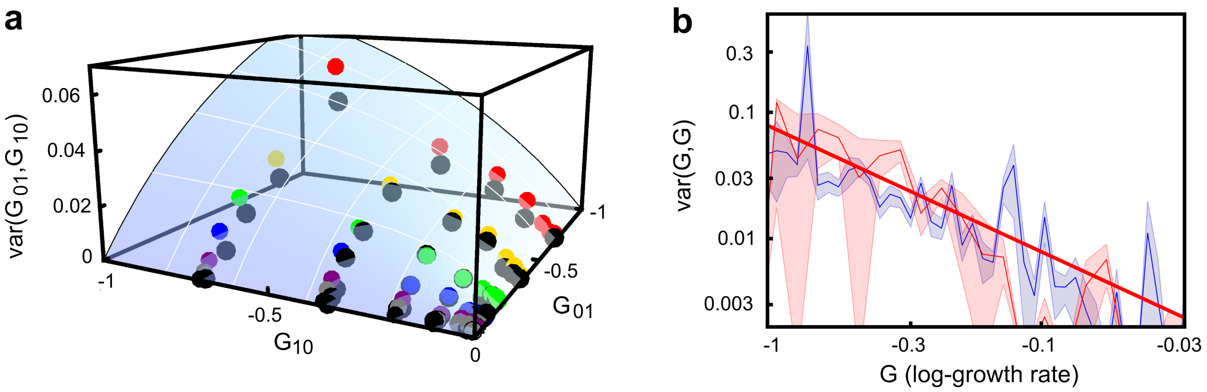

Figure S5 | DAmP "mutations" have similar epistatic interactions as entire gene knockouts. (a) Comparison between the epistasis observed in double gene knockout mutants (rainbow dots, same as in Fig. 2a) and the epistasis observed in mixed double mutants generated by combining a gene knockout and a DAmP (Decreased Abundance by mRNA Perturbation) perturbation of a different gene (black dots). (b) The red curve is the diagonal slice of the plot in (a) ($G_{01} = G_{10}$ within 20%, G = mean(G_{01}, G_{10})) and the red shaded area is the 25%-75% confidence interval for the mixed double mutants variance. For comparison it is superimposed to Figure 2c describing the variance for double gene knockouts (blue). As in Figure 2c the red line has equation var(G, G) = 0.079 G.

Figure S6

The Gene Ontology (GO) database collects annotations of eukaryotic genes and it can be exploited to compare the traditional and the geometric definitions of epistasis in their ability to identify interacting pairs. Two genes are dubbed as GO-interacting if the number of GO terms they share is larger than some threshold (specifically, if two genes have n and m GO terms, we consider the set of all genetic pairs with n and m GO terms and dub as "GO-interacting" the 5% pairs sharing the highest number of GO terms in that set).

The traditional definition of epistasis identifies slightly more GO-interacting pairs: among the 10,000 most interacting pairs according to the traditional definition 1308 are GO-interacting, whereas among the top 10,000 most interacting pairs according to the geometric definition 1210 are GO-interacting (Fig. S6a). Both definitions agree in identifying 1010 GO-interacting pairs; 515 GO-interacting pairs would be expected by picking 10,000 random gene pairs. The 298 pairs which are identified as interacting only by the traditional definition tend to involve genes with small growth rate effects, whereas the 200 pairs which are identified as interacting only by the geometric definition tend to involve genes with large growth defects. Importantly, when doubling the threshold rank discriminating between interacting and non-interacting pairs, the geometric definition discovers almost all of the interactions discovered by the traditional definition with the original threshold. In contrast, even when doubling the threshold rank, the traditional definition misses many of the interactions discovered by the geometric definition with the original threshold (Fig. S6b). The GO enrichment of these neglected pairs indicates that the traditional definition of epistasis may completely overlook important interactions between genes with large growth rate defects.

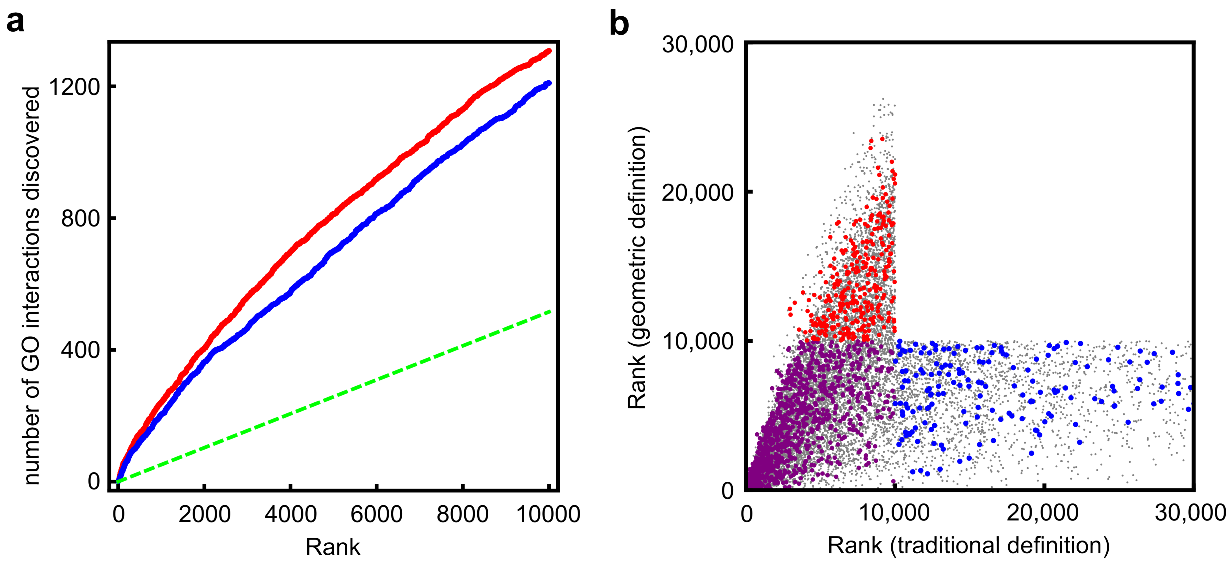

Figure S6 | Comparison between the traditional and the geometric definitions of epistasis. Each genetic pair is ranked according to the strength of the corresponding genetic interaction. (a) Number of Gene Ontology interactions discovered among the top-ranked pairs according to the geometric definition (blue), the traditional definition (red) and by selecting random pairs of genes (green dashed line). (b) Comparison of the ranks obtained by genetic pairs depending on how epistasis is defined. Each gray dot represent a genetic pair; larger dots represent interactions confirmed by the GO analysis (red: top-10,000 according to the traditional definition but not the geometric definition; blue: top-10,000 according to the geometric definition but not the traditional definition; purple: top-10,000 according to both definitions).

Supplementary Text 1

Rigorously introducing a new geometric model of epistasis compatible with all the features of the data is beyond the scope of this work. However it is worth providing some intuition for the experimentally observed dependence of the variance of epistasis:

$$\mathrm{var}\left(G_{01,}G_{10}\right) \; = \; 2\,c\,\frac{|G_{01}||G_{10}|}{|G_{01}|+|G_{10}|}$$

Let us consider two genetic backgrounds, *WT* (wild type) and *A* (wild type plus mutation *A*). A second mutation, *B*, is known to have an effect G_B in the wild type background. The unknown effect of mutation *B* in background *A* can be modeled as G_B + N(0, σ_B=Sqrt(2c G_B)). The first term represents a deterministic "drift" which embodies our previous knowledge about the effect of mutation *B* in the wild type background, whereas the "diffusion" term N(0, σ_B) represents the uncertainty due to epistasis. By following the path *WT* → *A* → *AB*, we would then predict for the double mutant *AB* a log growth rate of G_A + G_B + N(0, σ_B).

We could also consider *WT* and *B* (wild type plus mutation *B*) as the two original backgrounds. Mutation *A* has then an effect G_A in the wild type background and an effect G_A + N(0, σ_A=Sqrt(2c G_A)) in background *B*. In this case, by following the path *WT* → *B* → *AB*, we would predict for the double mutant *AB* a log growth rate of G_A + G_B + N(0, σ_A).

Each of the two routes provides some *information* about the log growth rate of the double mutant *AB*. Importantly, the mutant *AB* is the same regardless of which route is chosen (*WT* → *A* → *AB* or *WT* → *B* → *AB*). Because of this constraint[49], the log growth rate of *AB* can be estimated as G_{AB} = G_A + G_B + N(0, σ_{AB}), with N(0, σ_{AB}) proportional to N(0, σ_A) N(0, σ_B) and:

$$\sigma_{AB}^2 \; = \; \frac{\sigma_A^2\sigma_B^2}{\sigma_A^2+\sigma_B^2} \; = \; 2\mathrm{c}\,\frac{G_A\,G_B}{G_A+G_B} \; = \; \mathrm{var}\left(G_A,G_B\right)$$

Notably, the model has only one free parameter, the "diffusion constant" c, conceptually analogous to a fitness landscape's roughness.

Supplementary Text 2

The Fisher's geometric model is a simple model of epistasis in which epistatic interactions emerge from geometry rather than being introduced *ad hoc* through random variables and noise. Phenotypes are described by *d* quantitative traits which can assume any real value and a particular phenotype is represented by a point *x* in the *d*-dimensional space. A fitness value is associated to each point in such space. Since most naturally occurring populations are believed to be in proximity of a local fitness optimum (which we set at *x*=0), a common choice for the fitness function is: f(*x*) = - ½ *x* · *x*, where the dot denotes the scalar product. A wild type

organism with phenotype close to, but not exactly at the fitness optimum is represented by a vector $x_0 \neq 0$. It's fitness is then $f_0 = -\frac{1}{2}\, x_0 \cdot x_0$. A mutant is obtained by shifting the wild type phenotype in the *d*-dimensional space by a displacement d*x*. The fitness of the mutant is then $f(x_0+dx) = -\frac{1}{2}\,(x_0+dx) \cdot (x_0+dx) = -\frac{1}{2}\, x_0 \cdot x_0 - x_0 \cdot dx - \frac{1}{2}\, dx \cdot dx$, and the fitness effect of the mutation is $df = f(x_0+dx) - f(x_0) = -x_0 \cdot dx - \frac{1}{2}\, dx \cdot dx$. For small displacements df is approximately linear in $|dx|$, whereas for large displacements, or for displacements orthogonal to the fitness gradient, df is approximately proportional to $|dx|^2$.

Considering now two mutations and their combination in a double mutant:

$$df_1 = -x_0 \cdot dx_1 - \frac{1}{2}\, dx_1 \cdot dx_1$$

$$df_2 = -x_0 \cdot dx_2 - \frac{1}{2}\, dx_2 \cdot dx_2$$

$$df_{12} = -x_0 \cdot (dx_1+dx_2) - \frac{1}{2}\,(dx_1+dx_2) \cdot (dx_1+dx_2)$$

$$E = df_{12} - df_1 - df_2 = -dx_1 \cdot dx_2$$

Assuming that the displacements d*x* are isotropic in the *d*-dimensional space, the average of their scalar product vanishes. Fisher’s model predicts then that the mean epistasis is zero and that, on average, the effects of two mutations are additive. This observation points to the fact that the “fitness” values f in the Fisher's model are actually log-growth rates which, as we discussed in the main text, are on average additive variables.

In the Fisher's model , the standard deviation of epistatic interactions is:

$$\sigma_E^2 = \text{mean}(E^2) - \text{mean}(E)^2 = \text{mean}(\,(dx_1 \cdot dx_2)\,(dx_1 \cdot dx_2)\,) - 0 = |dx_1|^2\, |dx_2|^2\, \text{mean}(\cos^2(t))$$

The means are computed over all possible orientations of the displacement vectors dx_1 and dx_2 in the *d*-dimensional space, with t representing the angle between dx_1 and dx_2. Mean($\cos^2$(t)) is a *d*-dependent numerical factor that can be computed analytically. Using the fact that df can be approximately proportional to |dx| or to $|dx|^2$, the characteristic magnitude of the epistatic interaction $\sigma_E(df_1, df_2)$ can be proportional to $df_1\, df_2$ for small fitness effects and proportional to Sqrt(df_1 df_2) for large fitness effects. In particular, when $df_1 = df_2 = df$, σ_E(df, df) can be proportional to df^2 for small fitness effects and proportional to df for large fitness effects. As discussed in the main text, the experimentally observed dependence of the characteristic magnitude of the epistatic interactions with the log-growth effect of the mutations being combined is $\sigma_E(G, G) \propto$ Sqrt(G) and such dependence is weaker than the weakest dependence attainable in the Fisher’s model.

Supplementary Text 3

For millions of mutants the DRYGIN data set provides a growth rate g_{DRYGIN} and an estimate of the error on such growth rate σ_{DRYGIN}. Importantly, the value of "true" growth rate is not described by a normal distribution with mean g_{DRYGIN} and standard deviation σ_{DRYGIN}. This is because the experimental error is estimated from only four independent growth measurements. If the "true" growth rate of a mutant is g and the randomness intrinsic in the experiment leads to an uncertainty σ_g, each independent growth measurement is a random number extracted from a normal distribution $N(g, \sigma_g)$ with mean g and standard deviation σ_g. Importantly, the value of the "true" growth rate, as estimated after four measurements, is distributed according to a student's t-distribution with three degrees of freedom, not according to the original normal distribution $N(g, \sigma_g)$. For a very large number of measurements the t-distribution converges to the original normal distribution and the distinction is not important. However, with four measurements, the t-distribution has tails decaying as x^{-4} so that the difference between the estimated mean growth rate and the "true" growth rate can be much larger than what expected based upon the naïve assumption of an underlying normal distribution. Importantly, even if a t-distribution allows for larger fluctuations than a normal distribution, it's power-law tails are such that the variance remains finite, thus ensuring convergence for all the observables in the analysis carried out in the main text.

Supplementary Reference

1. Ernst MO (2010) Decisions made better. *Science* **329**:1022-1023
